# Broadband Dielectric Spectroscopy Study of Biobased Poly(alkylene 2,5-furanoate)s’ Molecular Dynamics

**DOI:** 10.3390/polym12061355

**Published:** 2020-06-16

**Authors:** Michelina Soccio, Daniel E. Martínez-Tong, Giulia Guidotti, Beatriz Robles-Hernández, Andrea Munari, Nadia Lotti, Angel Alegria

**Affiliations:** 1 Civil, Chemical, Environmental and Materials Engineering Department, University of Bologna, Via Terracini 28, 40131 Bologna, Italy; giulia.guidotti9@unibo.it (G.G.); andrea.munari@unibo.it (A.M.); nadia.lotti@unibo.it (N.L.); 2 Departamento de Física de Materiales, University of the Basque Country (UPV/EHU), P. Manuel Lardizábal 3, E-20018 San Sebastián, Spain; beatriz.robles@ehu.eus (B.R.-H.); Angel.Alegria@ehu.eus (A.A.); 3 Centro de Física de Materiales (CSIC–UPV/EHU), P. Manuel Lardizábal 5, E-20018 San Sebastián, Spain

**Keywords:** 2,5-furandicarboxylic acid, poly(2,5-alkylene furanoate)s, broadband dielectric spectroscopy, molecular dynamics, bio-based polymers

## Abstract

Poly(2,5-alkylene furanoate)s are bio-based, smart, and innovative polymers that are considered the most promising materials to replace oil-based plastics. These polymers can be synthesized using ecofriendly approaches, starting from renewable sources, and result into final products with properties comparable and even better than those presented by their terephthalic counterparts. In this work, we present the molecular dynamics of four 100% bio-based poly(alkylene 2,5-furanoate)s, using broadband dielectric spectroscopy measurements that covered a wide temperature and frequency range. We unveiled complex local relaxations, characterized by the simultaneous presence of two components, which were dependent on thermal treatment. The segmental relaxation showed relaxation times and strengths depending on the glycolic subunit length, which were furthermore confirmed by high-frequency experiments in the molten region of the polymers. Our results allowed determining structure–property relations that are able to provide further understanding about the excellent barrier properties of poly(alkylene 2,5-furanoate)s. In addition, we provide results of high industrial interest during polymer processing for possible industrial applications of poly(alkylene furanoate)s.

## 1. Introduction

The study of dynamic processes in polymers and soft matter systems provides a deep insight into the different role of the molecular constituents into the final materials’ properties. Broadband dielectric spectroscopy (BDS) is among the most well-established experimental techniques used for these studies, as shown by its extensive use in the past decades [1,2]. BDS allows probing the dipole reorientations and/or charge motions taking place within a sample when subjected to an AC electric field. The frequency (f) of the applied bias covers a wide range, from 10^10^ Hz to 10^−6^ Hz. At the same time, it is possible to control variables such as temperature and pressure. The application of BDS has been extended to perform simultaneous structural studies by its integration in big facilities as synchrotron and neutron scattering lines [3]. In addition, there have been several successful developments for the measurement of BDS at the nanoscale [4]. 

Recently, 2,5-furandicarboxylic acid (2,5-FDCA) has been recognized one of the most important 12 renewable building blocks selected by the US Department of Energy [5]. Its utilization in new bioplastics production has been considered a significant contribution to the sustainable development. In this framework, the class of 2,5-FDCA-based polyesters are of particular interest. Among them, poly(ethylene 2,5-furanoate) (PEF) is the most important and most studied member to date. Such polyester represents indeed the most credible bio-based alternative to poly(ethylene terephthalate) (PET) because of its properties, which are even superior to those of its terephthalic counterpart. Notable are the very good mechanical and the excellent gas barrier properties, which render this new polymer a very promising candidate in the food and beverage packaging [6,7,8,9,10,11,12,13,14,15,16]. More recently, the attention of researchers has been addressed on other polyesters of the family, specifically those containing a longer glycol subunit with respect to PEF. In fact, FDCA-based polyesters have been successfully synthesized starting from glycols with a number of C atoms ranging from 3 to 12. These new polyesters have been characterized from the thermal, mechanical, and dielectric point of view [17,18,19,20,21,22,23,24,25,26]. In fact, in the particular case of BDS, there have been several investigations focused on furan-based polymers, revealing complex molecular dynamics [27,28,29,30,31]. A recent work by Papamokos and collaborators presented a detailed study on the molecular dynamics of poly (alkylene 2,5-furanoates) using a combined approach of Density Functional Theory (DFT) calculations with BDS experiments [31]. The authors’ results revealed interesting conformational properties in these systems, driven by the packing of helical segments within the amorphous domains [31]. In addition, Guidotti et al. prepared by two-stage melt polycondensation four 100% bio-based polyesters of 2,5-furandicarboxylic acid, utilizing 1,3-propanediol, 1,4-butanediol, 1,5 pentanediol and 1,6-hexanediol (PTF, PBF, PPeF, and PHF, respectively) [24,26,28,32,33]. The four polyesters were successfully processed into films by compression molding, and such films were subjected to thermal, diffractometric, mechanical, and gas permeability characterization. The results indicated that glycol subunit length determined the final polymer properties, finding an odd–even trend [31,32]. In detail, the polymers with an odd number of carbon atoms in the glycol subunit showed a lower tendency to crystallize with consequent formation of a less perfect crystalline phase, i.e., they were characterized by a lower melting temperature than the polymers obtained from glycol with an even number of carbon atoms, and they exhibited superior gas barrier performances. In particular, poly(pentamethylene 2,5-furanoate) showed a very unusual and peculiar behavior, resulting in the best material in terms of barrier properties among those investigated and being characterized by a mechanical response typical of thermoplastic elastomers, despite its amorphous rubbery nature at room temperature [26,32].

In this view, in the present work, we describe the results obtained from a deep bulk BDS characterization of the four 100% bio-based poly(alkylene 2,5-furanoate)s previously synthesized. We studied the molecular dynamics of both fast-cooled samples, similar to those previously reported, as well as samples slow-cooled from the molten state, which might correspond to possible industrial processing conditions. In this latter case, we used a high-frequency BDS technique that allowed studying the molecular dynamics in the molten state. We aimed at addressing two goals. One is establishing clear structure–property relations, which are useful tools to explain the outstanding barrier properties exhibited by such polymers; the other is providing data of high industrial interest during polymer processing, thanks to the “design” of specific BDS experiments.

## 2. Materials and Methods 

### 2.1. Samples

**Scheme 1:**
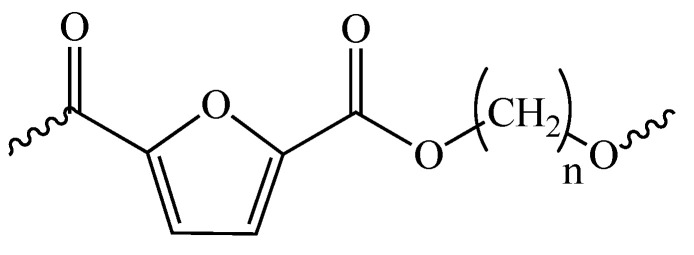
Chemical structure of the investigated poly(alkylene 2,5-furanoate)s. n refers to the number of methylene groups: 3, 4, 5 and 6 for PTF, PBF, PPeF and PHF, respectively.

The chemical structure of the poly(alkylene 2,5-furanoate) samples studied in this work, poly(trimethylene 2,5-furanoate) (PTF), poly(butylene 2,5-furanoate) (PBF), poly(pentamethylene 2,5-furanoate) (PPeF), and poly(hexamethylene 2,5-furanoate) (PHF) is presented in Scheme 1. The corresponding molecular and thermal characterization data are summarized in Table 1. The polyesters were synthesized through a two-stage solvent-free polycondensation process, as described elsewhere [24,26,28,32,33]. 

### 2.2. Broadband Dielectric Spectroscopy (BDS)

BDS measurements are performed using an impedance analyzer that allows the quantification of the complex electrical magnitudes as the impedance $Z^{*}\left(\omega \right)$, conductivity $\sigma^{*}\left(\omega \right)$, and dielectric permittivity $\varepsilon^{*}\left(\omega \right)$, among others. For all cases, ω=2πf. For the study of molecular dynamics in soft matter and polymers using BDS, the experimental results are usually presented using the complex dielectric permittivity function $\varepsilon^{*}\left(\omega \right)=\varepsilon^{\prime }\left(\omega \right)-i\varepsilon^{\prime \prime }\left(\omega \right)$. Here, $\varepsilon^{\prime }\left(\omega \right)$ is the real part of $\varepsilon^{*}\left(\omega \right)$, and it is related to relative permittivity of the sample, while $\varepsilon^{\prime \prime }\left(\omega \right)$ is the imaginary part of $\varepsilon^{*}\left(\omega \right)$, which represents the energy lost in the dielectric medium as the dipoles are oriented against random collisions. 

In this work, the BDS measurements were performed using a dielectric spectrometer (NOVOCONTROL Technologies GmbH & Co, Montabaur, Germany). The machine was equipped with an Alpha impedance analyzer (NOVOCONTROL Technologies), covering a frequency range between 1 MHz and 0.1 Hz. The temperature was controlled using a dry nitrogen jet (Quatro, by NOVOCONTROL Technologies), with a thermal stability better than ±0.5 K. We covered a temperature range between 183 and 453 K. Samples for BDS measurements were films of *c.a.* 150 μm in thickness, prepared by melt-pressing [26], and capped between Au-coated electrodes of 10 and 20 mm in diameter. We used PTFE spacers (150 μm thickness) to avoid any possible short circuits during measurements. 

We subjected all samples to two different thermal protocols. For a first case, we heated the samples from room temperature (approximately 300 K) to 453 K. This final temperature was selected to be above the melting/glass transition of all the studied polymers. We held the sample at 453 K for 5 min, and then, we rapidly cooled it by submerging the whole dielectric cell into a liquid nitrogen (LN_2_) reservoir. The sample was kept inside the LN_2_ for at least 10 min, while allowing cooling of the cryostat. Then, the dielectric cell was transferred back to the cryostat and BDS measurements started at 183 K, up to 453 K, at 5 K isothermal steps. From now on, the samples measured following this thermal treatment are called fast-cooled (FC) samples. For a second treatment, we heated the samples to 453 K and cooled them down to 183 K at 3 K/min within the cryostat. Then, we repeated the BDS measurements from 183 to 453 K at 5 K isothermal steps. In this case, we defined the samples measured using this protocol as slow-cooled (SL) samples.

Measurements performed in the high-frequency regime (10^6^–10^9^ Hz) were carried out using an E4991A RF-Impedance Analyzer (Agilent Technologies, Santa Barbara California, US). The samples were prepared by capping the polymers between brass electrodes of 10 mm in diameter. Polytetrafluoroethylene (PTFE) spacers (100 μm in thickness) were used to avoid possible short circuits. High-frequency measurements were performed from 453 to 303 K, using a 3 K/min ramp, measuring continuously.

## 3. Results

### 3.1. Local Dynamics of Poly(Alkylene 2,5-Furanoate)s (T « T_g_)

**Figure 1:**
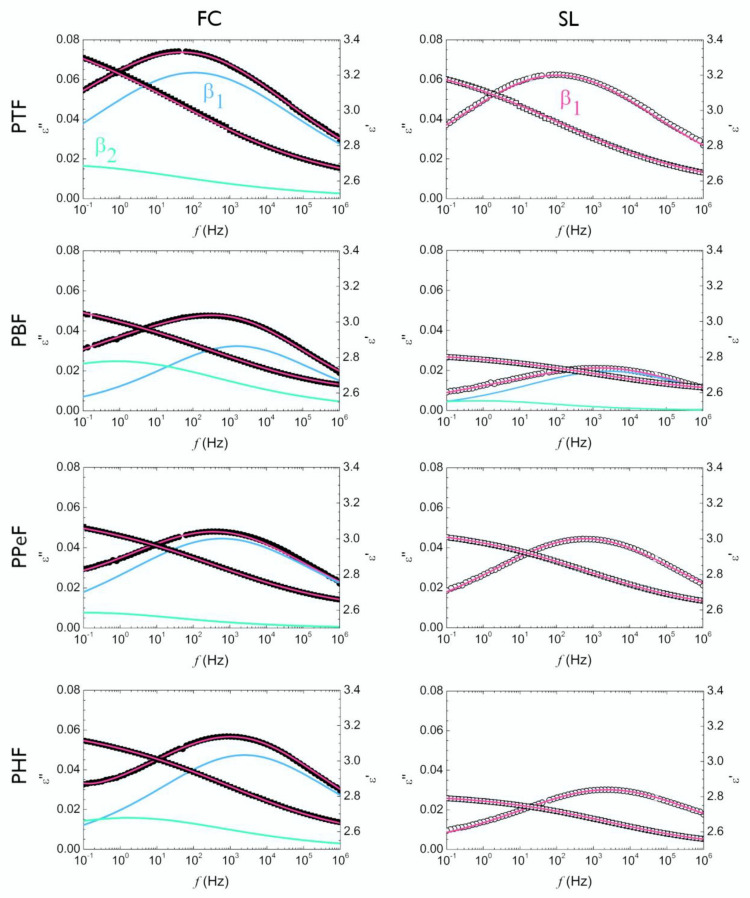
Broadband dielectric spectroscopy (BDS) results at 228 K. Circles correspond to ε″ and squares correspond to ε′. The fittings were carried out following Equation (1). Solid pink lines correspond to the total fit, while blue and green lines show the components of β_1_ and β_2_, respectively. The data corresponding for fast-cooled (FC)-PBF was adapted from Ref [28].

Figure 1 shows the real and imaginary components of $\varepsilon^{*}\left(\omega \right)$ at 228 K for each one of the samples and thermal treatments. This temperature was well below the glass transition (*T*_g_) of any of the presented polymers (see Table 1), and thus the relaxation processes should correspond to so-called local dynamics [1]. From Figure 1, we observed that as the glycolic subunit length of the poly(alkylene furanoate)s increased, the BDS signals shifted toward higher frequencies, indicating faster local dynamics. This result was observed to be independent from thermal treatments.

The shape and intensity of the dielectric relaxations showed interesting changes with thermal history. We observed that the FC samples presented asymmetric signals toward low frequencies, which is an evidence better resolved if looking at the $\varepsilon^{\prime \prime }\left(\omega \right)$ peaks. This asymmetry seemed to be reduced after slowly cooling from the melt, i.e., in the SL samples. This change was accompanied by a decrease in the relaxation strength (Δε), which is defined as the difference between the low and high-frequency limits of the $\varepsilon^{\prime }\left(\omega \right)$ spectra, or analogously by the area under the $\varepsilon^{\prime \prime }\left(\omega \right)$ peaks. The changes in shape and intensity were different between poly(alkylene furanoate)s with odd- and even-numbered glycolic subunits. On one hand, the samples with an odd C-number of glycolic subunits, PTF and PPeF, presented only a slight decrease in the relaxation strength after slow cooling. On the other hand, PBF and PHF showed distinct changes in Δε when comparing FC and SL treatments. In these last two polymers, there was a strong decrease in the relaxation strength of the SL samples, affecting the whole dielectric signal. Nonetheless, we noticed that the low-frequency part of the spectra was more influenced, as observed for the other polymers.

In order to evaluate these local relaxations, we fitted the $\varepsilon^{*}\left(\omega \right)$ data in the 213–273 K temperature range, to Cole–Cole (CC) functions described by [1]:

(1) $$\varepsilon^{*}\left(\omega \right)=\varepsilon_{\infty }+\frac{\Delta \varepsilon }{1+{\left(i\omega \tau_{CC}\right)}^{b}}$$

where $\varepsilon_{\infty }$ is the high-frequency limit of $\varepsilon^{\prime }\left(\omega \right)$, Δε is the relaxation strength, $\tau_{CC}$ is the relaxation time, and *b* is the shape parameter (symmetric broadening of the relaxation function). We observed that for a proper description of the BDS data for FC poly(alkylene furanoate)s, we required a sum of two Cole–Cole relaxations. Figure 1 shows the fitting results for PTF, PBF, PPeF, and PHF, at 228 K, by the different continuous lines. We highlight that the corresponding fits for FC PBF were taken from our previous work [28]. The faster component was called β_1_, and the slower one was called β_2_, which is in line with previous works [28,29,35]. However, for fitting SL samples, we observed that only a single Cole–Cole function was required for a good data description, with the exception of the PBF.

**Figure 2:**
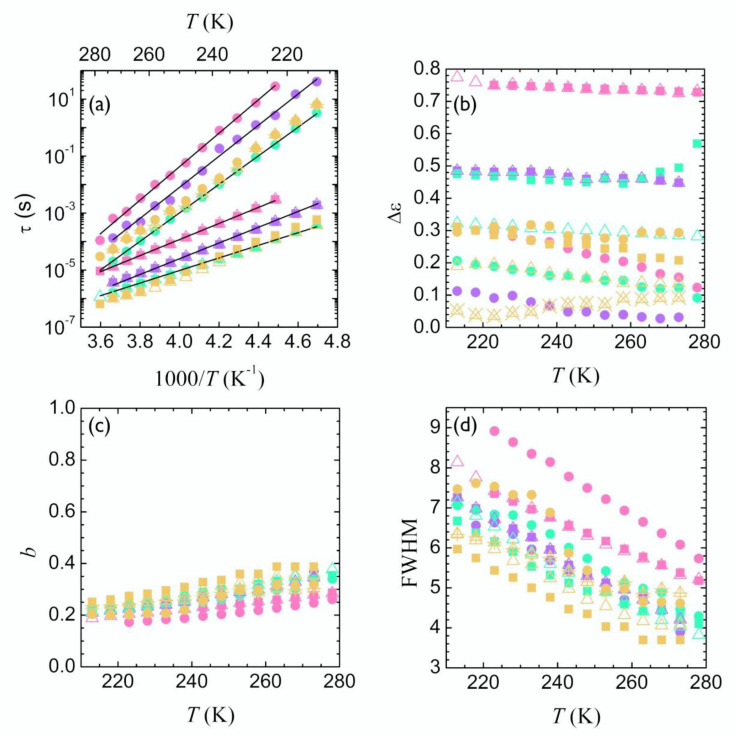
(**a**) Relaxation plot, (**b**,**c**) fitting parameters, and (**d**) full width at half maximum (FWHM) as a function of temperature for the local relaxations of poly(alkylene furanoate)s: PTF (light pink), PBF (yellow), PPeF (light purple), PHF (light green). Solid symbols correspond to the *β*_1_ (■) and *β*_2_ (●) contributions of FC samples, while empty symbols (Δ) correspond to the *β*_1_ contribution in SL samples. In the specific case of SL PBF, the contribution of *β*_2_ is shown by crossed triangles. The results for FC PBF were taken from Ref [28].

With the aim of providing the full picture of the local molecular dynamics, in Figure 2, we present the temperature dependence of fitting parameters obtained for all polymers and thermal treatments. In this figure, solid points correspond to FC samples while empty ones correspond to SL samples. Figure 2a shows the relaxation plot for all samples, where we have included also the results for PBF, taken from Ref [28]. In this relaxation plot, the data did not follow a trend according to the length of the glycolic subunit. We observed that the PTF and PPeF data laid closer to each other, while the PHF coincided with that previously reported for PBF. The relaxation times of the single CC function, describing the SL samples, coincided with those found for the β_1_ process of FC samples. All relaxations processes showed an Arrhenius behavior, modeled using the following Equation (1) [1]:

(2) $$\tau =\tau_{0}e^{\left(\frac{E}{RT}\right)}$$

where $\tau_{0}$ is a time constant, *E* is the activation energy, *R* is the gas constant, and *T* is the temperature. The continuous black lines in Figure 2a correspond to the fittings of the local processes to Equation (2). The obtained activation energies are summarized in Table 2. Please beware that the activation energies for the β_1_ processes of FC samples are the same as those found for the single CC of SL samples.

The values obtained for $E_{\beta 1}$ were constant within experimental errors for PTF, PBF, and PPeF. However, it decreased significantly for PHF, i.e., the polymer containing the longest glycolic subunit. The $E_{\beta 2}$ values were always larger, when compared to $E_{\beta 1}$, for each particular polymer. In addition, the polymers containing carbon-even-numbered glycolic subunits showed smaller $E_{\beta 2}$ activation energies.

Figure 2b shows the relaxation strength as a function of temperature. The $\Delta \varepsilon_{\beta_{1}}$ values obtained for FC samples were found to be temperature independent. PTF showed a value of almost 0.8, PPeF and PHF showed similar values around 0.5, and finally PBF presented a Δε close to 0.3. We observed the same $\Delta \varepsilon_{\beta_{1}}$ trend for SL samples, with the exception of PBF and PHF. In these cases, although the relaxation strength was also temperature independent, we quantified a decrease of about 35–40% in Δε. In the case of $\Delta \varepsilon_{\beta_{2}}$, the obtained results were fairly different. First, as already introduced, the *β*_2_ component was present only in FC samples, with the exception of PBF. This latter case is discussed in the Supporting Information. Second, the $\Delta \varepsilon_{\beta_{2}}$ values were smaller than those found for $\Delta \varepsilon_{\beta_{1}}$. Finally, we observed that $\Delta \varepsilon_{\beta_{2}}$ decreased with temperature for all samples. In addition, we noticed that the PTF and PBF $\Delta \varepsilon_{\beta_{2}}$ values were almost identical, while those for PHF and PPeF were progressively smaller. 

Finally, the CC shape parameter presented an increase with temperature from 0.2 to 0.4 (Figure 2c). Following the work by Schwartz and collaborators, we calculated the full width at half maximum (FWHM) of these local relaxations processes, using the CC shape parameter, as [36]:

(3) $$\mathrm{FWHM}=-0.516+\frac{1.058}{b}+\frac{0.039}{c}+\frac{0.563}{bc}$$

where in the specific case of the CC function, in Equation (3) c=1. As shown in Figure 2d, all the samples showed a decrease in the FWHM as the temperature increased. The broadest processes were shown by PTF, going even up to 9 decades in width. As the glycolic subunit of the polymer increased, the FWHM tended to decrease. For FC samples, we observed that the *β*_2_ contributions always showed wider processes than the *β*_1_, with the exception of PPeF, where the two components show fairly similar FWHM. The SL samples, where unique local process was found, presented widths similar to those found for *β*_1_.

### 3.2. Segmental Dynamics of Poly(Alkylene Furanoate)s (T ≥ T_g_)

**Figure 3:**
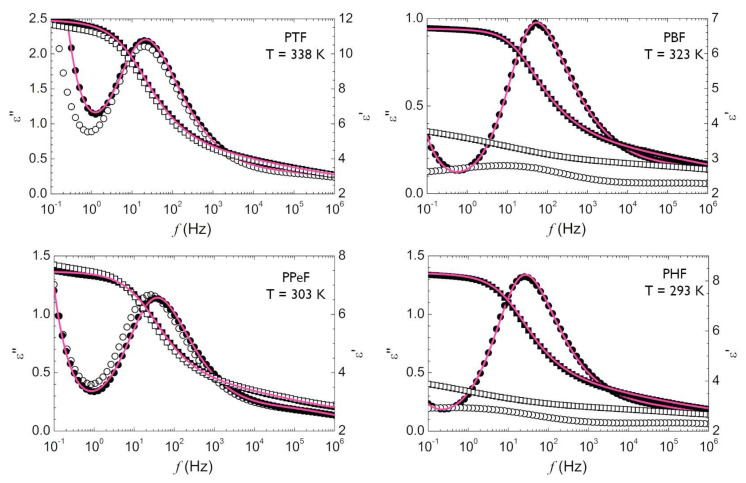
BDS results showing the segmental relaxation of the polymers. Circles correspond to ε″ and squares correspond to ε′. Solid symbols show the results for FC samples, while empty ones show the results for SL samples. Continuous lines correspond to fittings, as detailed in the main text. The data corresponding for FC PBF was adapted from Ref [28].

Figure 3 shows the BDS results for the different samples, at temperatures above *T*_g_, where the main relaxation phenomenon corresponds to the segmental motion of the polymer, which is also called alpha (α) relaxation. Since the glass transition of each of the polymers is different (Table 1), we selected a temperature where the α relaxation peak was in the frequency range between 10^1^ and 10^2^ Hz for all samples. After fast cooling from the melt, all polymers presented well-defined and intense dielectric relaxations. However, after slow cooling from the melt, we noticed interesting differences when comparing poly(alkylene furanoate)s with odd- and carbon-even-numbered glycolic subunits. The polyesters containing an odd number of methylene groups in the aliphatic segment, PTF and PPeF, showed almost no changes after fast/slow cooling from the melt. PTF presented a very small change in the relaxation strength, being lower for the SL sample, as readily observed in the $\varepsilon^{\prime }$ representation. The relaxation time, related to the position of the peak’s maximum in $\varepsilon^{\prime \prime }$, showed no differences. PPeF presented a slightly higher intensity and a small shift of the peak’s maximum toward lower frequencies in the SL case. The polymers with a carbon-even-numbered glycolic subunit, PBF and PHF, showed important changes comparing FC and SL samples. We noticed that in both cases, after slowly cooling from the melt, all the dynamics characteristics of the relaxation changed. In detail, for both polymers, we observed a dramatic decrease in the relaxation strength (approximately 50–60%) and a slowdown of the dynamics of about 1 decade (shift of the peaks’ maxima to lower frequencies). As carefully detailed in the following lines, the shape of the peaks changed as well.

In order to provide a full description of the segmental dynamics, we analyzed the BDS results in the 303–453 K temperature range. We focused on the dynamics of the segmental relaxation in the amorphous state only for the FC samples. For the physical description of the α relaxation, we used a Havriliak–Negami (HN) function, which is defined as [1]:

(4) $$\varepsilon^{*}\left(\omega \right)=\varepsilon_{\infty }+\frac{\Delta \varepsilon }{{\left(1+{\left(i\omega \tau_{\mathrm{HN}}\right)}^{b}\right)}^{c}}$$

where $\varepsilon_{\infty }$, Δε, and *b* have the same definitions as for Equation (1), while *c* represents the asymmetric broadening of the relaxation function (0 < *b, bc* ≤ 1). The relaxation time of the HN function ($\tau_{\mathrm{HN}}$) relates to the maximum of the dielectric losses ($\tau_{\mathrm{MAX}}$), by:

(5) τMAX=12πfMAX=τHN[sinbπ2+2c]−1b[sinbcπ2+2c]1b

where $f_{\mathrm{MAX}}$ is the frequency of maximum loss, and the rest are the HN function parameters. For properly fitting the segmental relaxation, we also included a contribution arising from the local process (now located at high frequencies), plus a direct current (DC) conductivity term ($\sigma_{\mathrm{DC}}$), described by: $\varepsilon^{*}\left(\omega \right)=\sigma_{\mathrm{DC}}/i\omega \varepsilon_{0}$. In addition, a single beta process was considered, phenomenologically. The local process relaxation time and dielectric strength were allowed to vary, while the shape was maintained constant and equal to the value obtained at 273 K.

**Figure 4:**
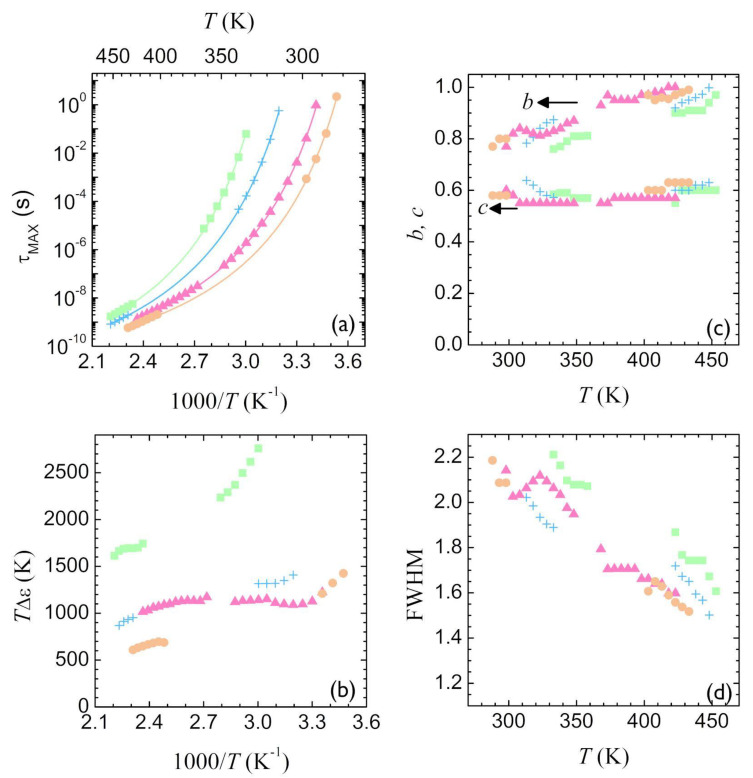
(**a**) Relaxation plot, (**b**,**c**) fitting parameters, and (**d**) FWHM, as a function of temperature for the segmental relaxations of FC poly(alkylene furanoate)s: ■ PTF, + PBF, ▲ PPeF, ● PHF. The low-temperature data for PBF (blue crosses) were adapted from Ref [28].

Figure 4 shows the obtained fitting parameters as a function of the temperature for all the fast-cooled polymers. In all panels of this figure, there are different temperatures without data points. This was related to the fact that PTF, PBF, and PHF suffered a cold crystallization process, which was detectable by our BDS experiments. Nevertheless, with the aim to provide further information about the poly(alkylene furanoate)s’ dynamics, we studied the segmental relaxation in the amorphous molten state, at temperatures above the melting point, using the high-frequency impedance analyzer (Figure S2). 

Figure 4a shows the relaxation plot of the α processes. All samples presented a non-Arrhenius behavior, modeled following the Vogel–Fulcher–Tammann (VFT) equation [1]:

(6) $$\tau_{\mathrm{MAX}}=\tau_{0}e^{\left(\frac{D\cdot T_{\mathrm{VFT}}}{T-T_{\mathrm{VFT}}}\right)}$$

where $\tau_{0}$ is a pre-exponential factor, *D* is a parameter related to the so-called dynamic fragility (deviation from the Arrhenius behavior), and $T_{\mathrm{VFT}}$ is the Vogel temperature. Table 3 summarizes the obtained results. We found that fixing the pre-exponential factor to a constant value of 10^−12^ s resulted in good fittings for all the samples. The obtained values of *D* were fairly similar, although one could envisage an increase of *D* as the glycolic subunit increases. The Vogel temperature showed a decrease as the glycolic subunit of the poly(alkylene furanoate)s was decreased. In addition, in Table 3, we present the dynamic glass transition temperature ($T_{g-\mathrm{BDS}}$) calculated using the VFT results as the temperature at which the relaxation time equals 100 s. 

For all samples, the relaxation strength (Δε) showed a decreasing trend as the temperature increased. In addition, Δε was dependent on the polymer chemical structure, being lower as the glycolic subunit length increased. The shape parameters presented similar values for all the studied samples. In all cases, the symmetric shape parameter (*b*) showed an increase as the temperature increased. At high enough temperatures, the parameter remained almost constant and close to the value of 1. The asymmetric shape parameter (*c*) was practically temperature independent, with values between 0.6 and 0.55. Using Equation (3), we calculated the FWHM for the segmental relaxation. We observed that all the α relaxation processes presented widths below 2 decades and became narrower as the temperature increased.

## 4. Discussion

### 4.1. Local Relaxations

The description of complex local relaxations as a sum of different contributions has been widely reported in the literature. Such sub-glass relaxations have been ascribed as due to the different conformations of simple σ bonds of the flexible moiety of the repeating unit [28,29,35,37,38]. For the specific case of furan-based polymers, previously we reported that the local relaxation of fast-cooled PBF was properly described by two Cole–Cole contributions. In that work [28], the faster component was interpreted as the manifestation of the dielectrically active O−C bond of the ester oxygen to the aliphatic carbon. The slower contribution was considered to originate from the C–CA link between the ester group carbon and the aromatic ring. Our current results indicated that all the studied samples that underwent fast cooling from the melt presented the contributions from these two movements. As concerning the *β*_1_ activation energies, one can see that they are similar for all the polymers, suggesting the glycol subunits have comparable mobility. The lowering of $E_{\beta 1}$ for PHF could be ascribed to the presence of a longer aliphatic segment favoring the dipole reorientations and charge motions associated to the O–C bonds. Likewise, the results regarding $E_{\beta 2}$ are also very interesting, evidencing a clear odd/even effect. In fact, although *β*_2_ originates from the C–CA bond present in all four polymers, the associated energy, $E_{\beta 2}$, is higher for the carbon-odd-numbered glycol containing polymers. The higher $E_{\beta 2}$ values for PTF and PPeF indicate a major hindering of the ring flipping in these polymers. A reduction of rotation of the furan ring around the ester groups could be related to their outstanding barrier performance.

After slowly cooling from the melt, we observed the presence of a “single” *β* process that, as a whole, was related to the faster local relaxation process (*β*_1_) of the FC samples. This indicated an intensity decrease of the slower component *β*_2_ that was associated to the C–CA bond rotation, which is very sensitive to thermal history. In fact, this result is quite interesting due to a number of reasons. First, previous reports on different poly(alkylene furanoate)s described the local relaxation as a single-component process, [27,29,30,31] whose activation energies were similar to those found for our *β*_1_ contributions. Since the presence of the *β*_2_ component depends on the thermal history of the samples, the absence of this relaxation in previous works can be linked to the different thermal protocols used. Second, we highlight that PBF was the only polymer that could not be well described using a single local process, after slow cooling from the melt (see Supporting Information). This result is in line with our previous report dealing with the molecular dynamics of PBF: in that paper, we indeed reported a multi-component local relaxation not only for the fast-cooled sample, but also for a semicrystalline one [28].

Regarding the relaxation strength, as one can see from the results reported for the FC and SL samples, a general Δε decrease of the local relaxation process in the SL materials can be evidenced, in which the reduction was more pronounced for the C-even-numbered glycol-containing polymers, PBF and PHF, which were able to crystallize under the experimental conditions adopted [31,32]. In fact, as previously reported, the relaxation strength of local motions can be deeply affected by crystallinity [28,31]. In our present work, the effect of crystal development in poly(butylene 2,5-furanoate) and poly(hexamethylene 2,5-furanoate) affected both *β*_1_ and *β*_2_. The results obtained for PBF indicated that both local components were still detectable after cold crystallization, as previously reported. However in PHF, the *β*_2_ component was affected to a higher extent, indicating that in this polymer, the C–CA bond rotation was more influenced by crystal development [28].

Concerning the C-odd-numbered glycol moiety polymers, PTF and PPeF, very interesting results have been obtained. As evidenced by calorimetric and diffractometric analyses, both poly(trimethylene 2,5-furanoate) and poly(pentamethylene 2,5-furanoate) do not undergo crystallization even under slow cooling (3 K/min) from the melt [24,26]; nevertheless, a Δε decrease of the *β*_2_ component has been detected. The lowering of the *β*_2_ process in the SL PTF and PPeF suggests a reduction of the C–CA bond mobility even if the crystalline phase does not develop. This effect could be explained considering the establishment of inter-chain interactions, as hydrogen bonds and π/π stackings [16,26,31,32], which ultimately hinder the furan ring flipping around the C–CA bonds.

Finally, the last of the dynamics characteristics, the shape parameter, allowed calculating the FWHM of the CC functions. We obtained a broad distribution of relaxation times, which was in line with recent BDS reports on poly(alkylene furanoate)s [31].

### 4.2. Segmental Relaxation

The dielectric segmental relaxation also showed differences between the two thermal histories (Figure 3). In the case of FC samples, the temperature evolution of the dielectric segmental relaxation allowed observing a cold crystallization phenomenon, taking place upon heating once the glass-to-rubber transition was exceeded. In this case, there was not any odd/even effect, since all the materials showed a cold crystallization, with the exception of PPeF. This result is in line with previous works where PTF, PBF, and PHF showed a cold crystallization [24,28,31,32,33], while PPeF remained in an amorphous state regardless of the thermal treatments [26,31,32]. In fact, this particular material only showed crystallization after being stored at room temperature for over 6 months [39,40]. On the contrary, in the case of SL samples, the melt crystallization occurring while slowly cooling showed an odd/even effect. For PBF and PHF, we detected a strong change in the relaxation intensity and slowdown of the dynamics (Figure 3). However, the polyesters containing carbon-odd-numbered glycolic subunits did not present remarkable changes in the α relaxation, which is in line with local relaxation results. The slight changes in SL PTF and PPeF segmental dynamics could be related to possible inter-chain interactions, also affecting the local dynamics.

In the present work, the analysis of the segmental relaxation was focused exclusively on amorphous poly(alkylene furanoate)s, using the BDS data collected after FC and in the molten state. The VFT law describing the temperature dependence of the segmental relaxation times presented interesting differences comparing with previous literature reports. First, we obtained a pre-exponential factor of 10^−12^ s, which differs from the so-far widely used (10^−14^ s) in the analysis of poly(alkylene furanoate)s [27,30,31,33]. This difference can be related to the addition in our present report of high-frequency data, allowing a “more complete” description of the segmental dynamics. This finding resulted in significant changes of the *D* parameter with respect to most of the values previously reported [28,29,31]. From the current analysis, we obtained values of *D* in the range of 4.5 to 5.1, presenting an increasing trend with the length of the glycolic subunit. Regarding the Vogel temperature, as well as the $T_{g-\mathrm{BDS}}$, we obtained values fairly close to those of the calorimetric *T*_g_s (Table 1), evidencing the higher macromolecular mobility with increasing aliphatic subunit length. In addition, our results were comparable to those previously reported [28,29,31]. The only sample showing a marked difference between $T_{g-\mathrm{BDS}}$ and $T_{g}$ (calorimetric) values was PHF, in which the glass-to-rubber transition determined by DSC analysis was much higher than the value calculated by the BDS technique. This difference can be attributed to the semicrystalline nature of the sample measured by DSC [32] and, consequently to the mobility restriction effect exerted by the crystals. 

Using the VFT data, we calculated the so-called dynamic fragility (*m*), which is a parameter related to steepness of τ(T) in the vicinity of $T_{g}$, and that quantifies the deviation of a VFT law from an Arrhenius behavior. The steepness index is defined as [41]:

(7) $$m={\frac{d\log\;\tau }{d\left(\frac{T_{g}}{T}\right)}|}_{T=T_{g}}$$

Using the VFT law (Equation (3)), the above equation transforms to [42]:

(8) $$m=\frac{D\cdot T_{\mathrm{VFT}}\cdot T_{g}}{\ln10\cdot {\left(T_{g}-T_{\mathrm{VFT}}\right)}^{2}}$$

The obtained results for *m*, by means of Equation (6), are summarized in Table 2. We found relatively similar dynamic fragility values for our set of poly(alkylene furanoate)s, with a general decreasing trend with the glycol subunit length. As known, fragility is associated with inter-/intra-molecular interactions, in which *m* is a measure of the bond interactions during the vitrification of glass-forming liquids [43]. Typically, the higher the attractive interactions, the higher the fragility. In this view, the greater *m* values for PTF could be due to the highest furan moiety number per unit chain length, being the aromatic ring responsible for the establishment of hydrogen bonds and π/π stackings. The *m* values here reported, although slightly higher, are in line with the previous report by Papamokos and collaborators [31], where the authors found that the amorphous samples’ fragility is fairly independent of the aliphatic segment length.

The temperature dependence of the segmental relaxation strength (Figure 4b) showed a decrease with temperature, which is evident for PTF, PBF, and PHF, whereas it is hardly detectable for PPeF. These results are in agreement with previous reports [28,29,31,39]. This difference could be explained as due to the dipole orientation during the chain ordering, for the cold crystallizing polymers, i.e., PTF, PBF, and PHF, whereas PPeF does not undergo cold crystallization. The high-frequency data at high temperatures, where the polymers are all in the molten state, showed a similar trend. Concerning the relaxation shape, we found quite narrow α relaxation peaks for all the samples, *b* parameter being around 0.8 at low temperatures, indicating a sharp distribution of the relaxation times in the amorphous state further narrowing as the temperature rises (*b* increasing). As concerns the symmetry parameter, *c*, it is very similar for all the furan-based samples (≈ 0.5–0.6) and typical of amorphous polyesters. Finally, no effect of temperature has been observed for *c*.

**Figure 5:**
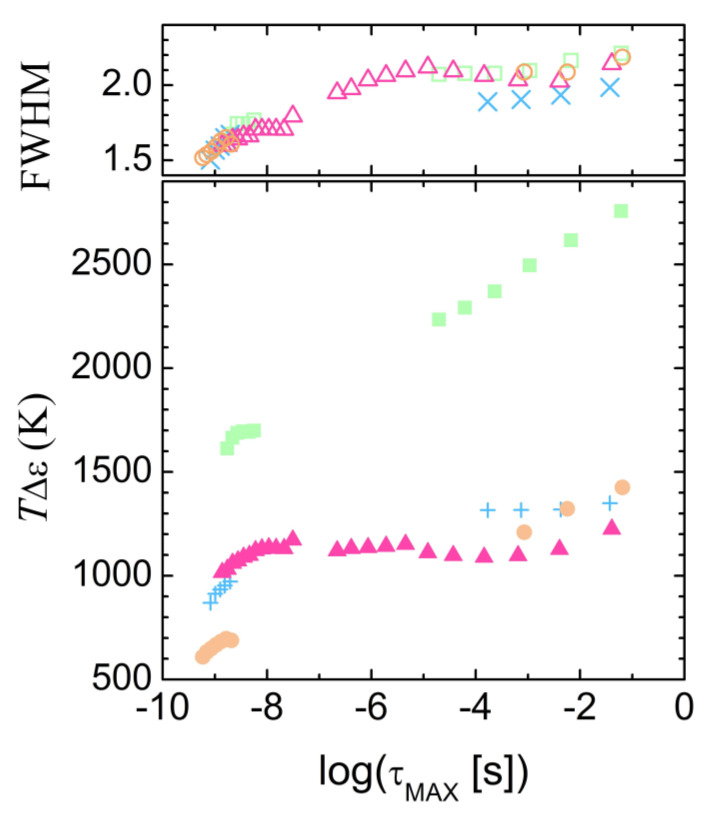
Relaxation strength (TΔε) and FWHM as a function of the relaxation time ($\tau_{\mathrm{MAX}}$). Solid symbols correspond to: ■ PTF, + PBF, ▲ PPeF, ● PHF. Empty symbols show the FWHM data: □ PTF, × PBF, Δ PPeF, ○ PHF.

Considering that the studied poly(alkylene furanoate)s have different segmental rates, in Figure 5, we show the segmental relaxation strength (TΔε) and FWHM as a function of the main relaxation time ($\tau_{\mathrm{MAX}}$). This useful representation allows a deeper analysis of the changes of the strength and shape of the α relaxation, among the presented polymers. Figure 5 clearly shows that the FWHM (open symbols) does not depend much on the glycol subunit length, with values typically observed in amorphous polymers. On the contrary, the relaxation strength (filled symbols) showed values clearly higher for PTF, while the rest of the materials presented comparable results. However, for PHF, we observed that at high frequencies, the TΔε values significantly decreased. These results could suggest an odd/even effect on the Δε values. 

## 5. Conclusions

We have studied the local and segmental molecular dynamics of the poly(alkylene furanoate) family by broadband dielectric spectroscopy. The experiments were performed on samples subjected to two different thermal treatments, in particular, fast and slow cooling from the molten state. These thermal protocols are of interest when considering possible industrial and practical applications. After fast cooling, the BDS experiments allowed determining the local dielectric relaxation of all polymers in the amorphous state. In addition, we also were able to analyze the segmental dynamics in the amorphous state, in proximity of the glass transition. Slow cooling from the melt resulted in two different structural states. The poly(alkylene furanoate)s containing carbon-odd-numbered glycolic subunits remained amorphous, while those with an even number of methylene groups underwent crystallization. Besides the changes associated to the crystallization process, which have been already discussed deeply by the scientific community, we highlight the following results. We found a bimodal local relaxation for all the polymers, being the two components differently affected by the kind of thermal treatment. The slower local motion, related to the C–CA group linking the furan ring and the aliphatic segment, turned out to be particularly decreased after slow cooling. This reduced mobility could be of relevance for the gas transport properties of the poly(alkylene furanoate)s films.

The segmental relaxation showed a more conventional behavior, where the semicrystalline samples presented weaker and more complex relaxation processes. Comparing the results of samples in the amorphous state, we found relaxation times and relaxation strengths depending on the glycolic subunit length. This result was confirmed with experiments exploring the high-temperature region, where the polymers were all in the molten state. Interestingly, the molten state would correspond to most of the processing conditions, and then our results are of relevance for possible industrial applications of poly(alkylene furanoate)s.

Finally, we highlight that in the specific case of PPeF, intriguing results have been found. Particularly, there are significant differences in the α relaxation range, depending on the thermal treatment unlike PTF, despite both remaining in the amorphous state after slow cooling. The unique and special behavior of PPeF has been object of detailed study in a recent work [39].

## Figures and Tables

**Table 1:** Molecular and thermal characterization data of the poly(alkylene 2,5-furanoate) samples studied.

Sample	*M*_n_ (kg/mol)	PDI	*T*_g_ (K)	*T*_m_ (K)
PTF [24]	30	2.3	325	442
PBF [28,33]	36.1 *	-	308	437
PPeF [26]	29.6	2.4	286	-
PHF [32]	28.9	2.3	286	414

* calculated from intrinsic viscosity values [34].

**Table 2:** Activation energies (E) of local relaxation processes for poly(alkylene furanoate)s.

Sample	PTF kJ/mol	PBF (FC) [6] kJ/mol	PPeF kJ/mol	PHF kJ/mol
$E_{\beta 1}$	52 ± 1	50 ± 2	52 ± 1	42 ± 1
$E_{\beta 2}$	112 ± 2	89 ± 1	104 ± 2	96 ± 2

**Table 3:** VFT parameters obtained for the α-relaxation of poly(alkylene furanoate)s.

Sample	PTF	PBF	PPeF	PHF
$\tau_{0}$ (s)	10^−12^ (fixed)			
*D*	4.5 ± 0.1	4.8 ± 0.1	5.1 ± 0.06	5.0 ± 0.1
$T_{\mathrm{VFT}}$ (K)	282 ± 1	266 ± 1	247 ± 1	240 ± 1
$T_{g-\mathrm{BDS}}$ (K)	321 ± 1	306 ± 1	286 ± 1	277 ± 1
*m*	116 ± 3	106 ± 3	103 ± 3	105 ± 3

## Supplementary Materials

The following are available online at https://www.mdpi.com/2073-4360/12/6/1355/s1, Figure S1: BDS data and fits for SL PBF, Figure S2: BDS high frequency data for PTF, PBF, PPeF, PHF.
