# Supplementary material for: Broadband Dielectric Spectroscopy Study of Biobased Poly(alkylene 2,5-furanoate)s’ Molecular Dynamics
Source: Polymers (Basel). 2020 Jun 16;12(6):1355. doi: 10.3390/polym12061355 (PMC7361705; doi:10.3390/polym12061355)
Supplement: Supplementary file 1 [file polymers-12-01355-s001.pdf]

**Supplementary Materials**

**PBF Fitting.** In order to provide comparable data, in the current work, we also studied the PBF molecular dynamics after slowly cooling from the melt at 3 K/min. We found that the best fit was obtained when using two CC contributions, which is in line with the previous report. In fact, when using only a single CC component, we noticed that not only was the data description the worst, but also that the maximum of the peak and thus the relaxation time did not coincide with that of $\beta_1$ for the FC case. We quantified a $\tau(\beta_1)$ of $6.03\cdot10^{-5}$ s, which increased to $1.81\cdot10^{-4}$ s when using a single CC component. This result is carefully shown in Figure S1.

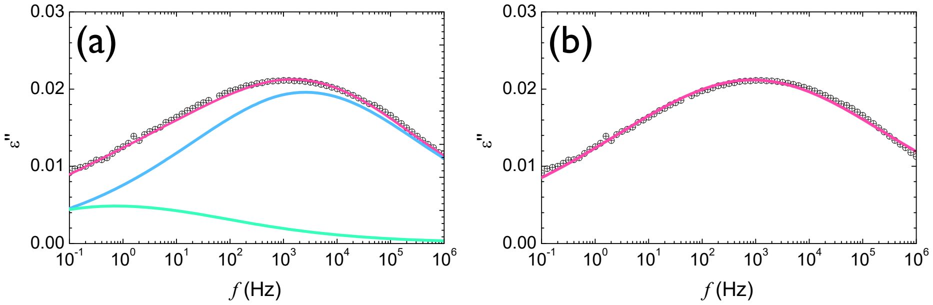


**Figure S1.** BDS data (points) and fits (lines) for SL PBF. Left panel shows the results using two CC functions and right panel the results using a single CC function.

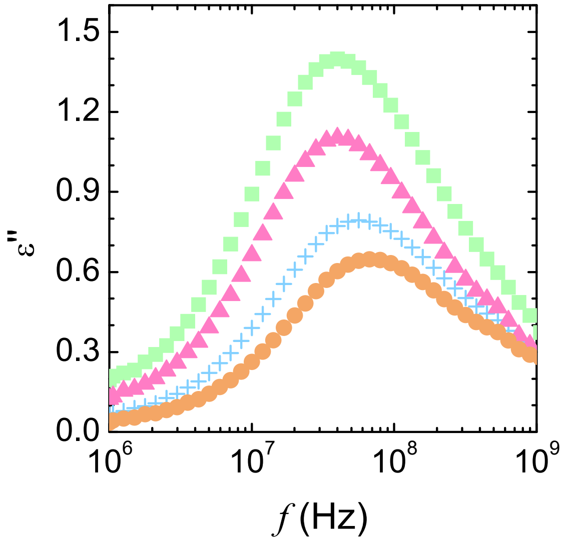


**Figure S2.** BDS high-frequency data for ■ PTF (438 K), + PBF (428 K), ▲ PPeF (403 K), ● PHF (403 K).
